# Machine learning techniques to predict different levels of hospital care of CoVid-19

**DOI:** 10.1007/s10489-021-02743-2

**Published:** 2021-09-10

**Authors:** Elena Hernández-Pereira, Oscar Fontenla-Romero, Verónica Bolón-Canedo, Brais Cancela-Barizo, Bertha Guijarro-Berdiñas, Amparo Alonso-Betanzos

**Affiliations:** Universidade da Coruña. CITIC Research and Development Laboratory in Artificial Intelligence (LIDIA) Facultad de informática, Campus de Elviña s/n. A, Coruña, Spain

**Keywords:** CoVid-19, Machine learning, Supervised classification, Feature selection

## Abstract

In this study, we analyze the capability of several state of the art machine learning methods to predict whether patients diagnosed with CoVid-19 (CoronaVirus disease 2019) will need different levels of hospital care assistance (regular hospital admission or intensive care unit admission), during the course of their illness, using only demographic and clinical data. For this research, a data set of 10,454 patients from 14 hospitals in Galicia (Spain) was used. Each patient is characterized by 833 variables, two of which are age and gender and the other are records of diseases or conditions in their medical history. In addition, for each patient, his/her history of hospital or intensive care unit (ICU) admissions due to CoVid-19 is available. This clinical history will serve to label each patient and thus being able to assess the predictions of the model. Our aim is to identify which model delivers the best accuracies for both hospital and ICU admissions only using demographic variables and some structured clinical data, as well as identifying which of those are more relevant in both cases. The results obtained in the experimental study show that the best models are those based on oversampling as a preprocessing phase to balance the distribution of classes. Using these models and all the available features, we achieved an area under the curve (AUC) of 76.1% and 80.4% for predicting the need of hospital and ICU admissions, respectively. Furthermore, feature selection and oversampling techniques were applied and it has been experimentally verified that the relevant variables for the classification are age and gender, since only using these two features the performance of the models is not degraded for the two mentioned prediction problems.

## Introduction

Coronavirus disease 2019 (CoVid-19), caused by severe acute respiratory syndrome coronavirus 2 (SARS-CoV-2), has become an unprecedented public health crisis, being declared by the World Health Organization as a pandemic in March 2020. The so-called first wave killed almost 700,000 people before the summer of this year. Since then, there have been other waves that appear to be even worse in the number of people affected and, in some cases, even in the number of deaths. The clinical symptoms of patients infected with CoVid-19 include, among others, fever, cough, shortness of breath, myalgia, fatigue, decrease of leukomonocyte and abnormal chest computed tomography imaging. The process of treatment and cure of the disease can be carried out by the patient at home or at some point it may require admission to the hospital or even to the intensive care unit (ICU). Because of this, the Health Systems of many countries in the world have reached the point of collapse due to the increasing and simultaneously demand for ICUs. For this reason national governments are trying to apply restrictive policies so as to avoid new collapses of the hospitals. Therefore, it has become urgent to identify and predict those cases that could progress to critical due to the high mortality rate of the latter. In order to make these predictions, Artificial Intelligence models can be used. Among them, Machine Learning (ML) algorithms are specially useful as nowadays we are able to generate and collect an impressive amount of data, that can be later used for further analysis. This is especially important in the field of Medicine, in which several Artificial Intelligence models have been used to automatize time-consuming and subjective manual tasks performed by practitioners. In particular, machine learning techniques have been widely and successfully applied to medical diagnosis for decades [11, 17].

In this research, we analyzed the capability of automatic classification systems from the field of machine learning to identify, based on data from patients diagnosed with CoVid-19, those cases that will require either hospital or intensive care admission at some point in their illness. Our aim is to find out which ML method and variables are the best predictors for both modes of hospital admissions. Up to our knowledge, this is the first work that addresses these two important issues at a time using only clinical and demographic data. The development of these models can be a very useful tool for planning future needs in hospital management based on the cases detected.

## Related work

Since the very beginning of the CoVid-19 outbreak, machine learning researchers have made a huge effort to search for new algorithms to support in tackle this pandemic. Lalmuanawma et al. [19] reviewed the latest advances of machine learning to improve treatment, medication, screening, prediction, forecasting, contact tracing, and drug/vaccine development process for the CoVid-19 pandemic. Tseng et al. [32] made a survey summarizing several different applications of computational intelligence techniques for confronting different problems within the CoVid-19 pandemia, namely: (i) tracking and predicting virus propagation, such as in Cassaro et al., Kucharski et al. and Salgotra et al. [10, 18, 30] (ii) characterization of the virus infections, as in the works by Oh et al. and Roy et al. [25, 29] (iii) design of adequate treatments as in Ren et al. [27], (iv) development of precaution mechanisms, approached in the works by Niazkar et al. and Salgotra et al. [24, 30] and finally, (v) public health policy making, using genetic algorithms [38], or fuzzy logic [36].

Our proposal belongs to the type (ii) characterization of the virus infections, in which we aim at risk-profiling and prediction of the evolution of the patients, specifically we are interested in hospitalization and ICU transfer predictions. Regarding this task, a few works can be found in the literature. Some of these are related with a general prediction of ICU and hospitalization loads, but without referring to specific patients, as for example is the case of Ritter et al. [28], in which a probabilistic model is applied to 3 different regions in Europe (Lombardia, Madrid and Berlin). More recently, broader studies such as the one in Bergman et al. [3] over the Sweden population (including health and CoVid patients), had tried to predict risk factors for CoVid diagnosis, hospitalization-ICU and non-ICU, and mortality. In this study the authors have found that older age, male sex, and comorbidity in general are risk factors for COVID-19 hospitalization and, with the exception of male sex, risk factors for COVID-19 diagnosis without hospitalization. Regarding the identification of ICU/hospitalization for each patient, in the work by Prytherch et al. [26], a modified version of the Early Warning Score (EWS) was used over a small data set consisting of 36 consecutive PCR-positive CoVid-19 patients admitted to the medical wards of a hospital, concluding that median EWS was significantly higher in a time-dependent manner in the ICU group than in the non-ICU group. However simple, the tool might help clinicians in the triage in the emergency services of the hospital, detecting in advance CoVid-19 patients who will require ICU admission. Even so, the study is quite limited as the number of patients is low, and only one center has been considered. In the work by Zhao et al. [39], a study of the features that could predict ICU admission and mortality for 641 hospitalized CoVid-19 confirmed patients was carried out, identifying 5 variables for the ICU case by using Logistic regression, but the study is restricted to only one big hospital. In Yitao et al. [37] the study is centered on finding the best predictors for clinical deterioration in patients admitted to the hospital with a confirmed diagnosis of pneumonia caused by COVID-19. The study was carried out in a center in China with a small sample of 257 patients.

Another set of studies have employed Deep Learning to analyze images for COVID-19 diagnosis and patient triage, using chest-X Ray images [25], or lung ultrasound images [29], but as it is has been extremely difficult to collect large set of well-curated images for training neural networks, the results although encouraging are quite restricted.

Regarding the prediction of hospitalization and ICU transfer, there are few papers available analyzing slightly more than a thousand of patients. In the work by Nemati et al. [23], the authors have applied several statistical and machine-learning models to predict the patient discharge time from the hospital, using a data set of 1,182 patients, and found out that sex and age are most accurate discharge-time predictors, although again the small size of the data set prevent them for generalization. Cheng et al. [8] have specifically dealt with the prediction of discharge time and ICU transfer in already hospitalized CoVid-19 patients. This latter study used a data set of 1,987 patients already admitted to one specific hospital, employing a Random Forest to predict the need for ICU transfer. Although the obtained results are similar to our proposal, we introduce some improvements: our data set is considerably larger; we use a feature selection algorithm to identify the relevant features (instead of using all available features for each patient), and we have studied a complete geographical area with 4 different hospital areas, including several referral and small hospitals. Besides, we have aimed also at studying different machine learning techniques with different representative feature subsets. Finally, in the work presented by Schwab et al. [31] they developed and evaluated clinical predictive models for CoVid-19 that estimate the likelihood of a positive SARS-CoV-2 test in patients presenting at hospitals, also providing the likelihood of hospital and ICU admission in patients who are SARS-CoV-2 positive. They evaluated the developed clinical predictive models in a retrospective evaluation using a cohort of 5,644 hospital patients in only one hospital in São Paulo, Brazil. In that study, in addition to patient demographic and clinical data, blood test data is also used, which requires an additional cost of blood testing that is not widely available to the entire population.

Concerning CoVid-19 forecasting, several ML approaches have been proposed. Mehta et al. [21] develop county-level prediction around near future disease movement for CoVid-19 occurrences using publicly available data. The model predictions showed a sensitivity over 71% and specificity over 94% for models built using data from March 14 to 31, 2020. It was found that population, population density, percentage of people aged > 70 years, and prevalence of comorbidities play an important role in predicting CoVid-19 occurrences. Also, they observed a positive association at the county level between urbanicity and vulnerability to CoVid-19. Watson et al. [34] embed a Bayesian time series model and a random forest algorithm within an epidemiological compartmental model which predicts deaths using COVID-19 data and population-level characteristics in U.S. states. The model was evaluated by training it on progressively longer periods of the pandemic and computing its predictive accuracy over 21-day forecasts. The accuracy of this CoVid-19 model offer reliable predictions and uncertainty estimates for the current trajectory of the pandemic in the U.S.. Chowell and Luo [9] propose and assess the performance of two ensemble modeling schemes with different parametric bootstrapping procedures for trajectory forecasting and uncertainty quantification. The performance of the methods are demonstrated using a diversity of epidemic datasets, including CoVid-19. The ensemble method that randomly selects a model from the set of individual models for each time point of the trajectory of the epidemic frequently outcompeted the individual models achieving not only better coverage rate of the 95% prediction interval but also improved mean interval scores across a diversity of the epidemic datasets. Finally, in Ahmed et al. [1] proposed an ensemble of feed-forward neural networks to forecast CoVid-19 outbreak in Iraq. This study highlights some key questions about this pandemic using data analytics. Forecasting were achieved with accuracy of 87.6% for daily infections, 82.4% for daily recovered cases, and 84.3% for daily deaths.

## Problems to be addressed and data set

Using data from the medical history of patients, this work develops an automatic method to address the following two classification problems, which could be of interest as an aid to decision-making in planning medical resources:

1. Will a patient diagnosed with CoVid-19 need to be hospitalized at some point in the disease process? This would allow us to determine the possibility that a incipiently diagnosed patient may need to be admitted to a hospital in the future.
2. Will a patient diagnosed with CoVid-19 need to be admitted to the ICU at some point in the disease process? This would allow us to determine the possibility that an incipiently diagnosed patient will potentially need intensive care attention in the future.

**Fig. 1:**
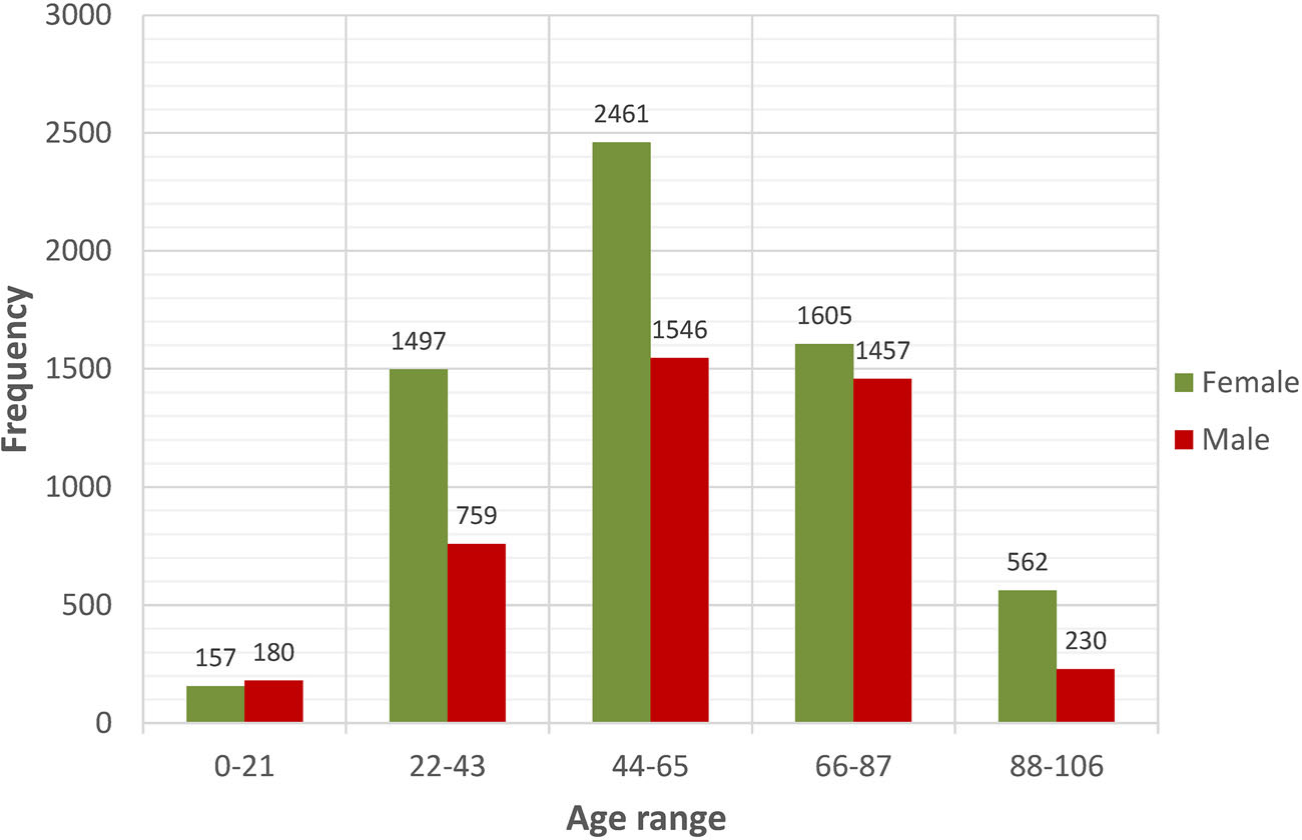
Age distribution of the 10,454 patients according to gender

To carry out this research study, we have used a data set containing 10,454 patients diagnosed with CoVid-19 in Galicia. Located in the northwestern of Spain, with 29.575 *km*^2^, it is the 5th region in terms of population with 2.698.764 inhabitants. It has a density of 91 inhabitants per *km*^2^, very similar to the population density of Spain. The admitted patients for the study are from 14 hospitals throughout the region. Each patient is characterized by 833 variables, like age and gender, together with another 831 variables representing the patient’s medical history. The latter are binary variables describing whether or not the patient has had any type of medical condition, such as hypertension, asthma or diabetes. Figure 1 shows the distribution of patients in 5 age ranges grouped by sex. These data were collected from March to May, 2020, although, for each patient, the history of hospital or intensive care unit (ICU) admissions due to CoVid-19 is available from December 23, 2019 until May 9, 2020.

To address the two classification problems, each patient in the data set was labeled analyzing the admission history in both hospital or ICU. For the first case, all patients who were hospitalized at some point (room or ICU) were labeled with a class 1 and the rest with a class 0. Performing this labeling, 3,024 patients were labeled as class 1, representing 28.9% of the total, and 7,430 as class 0, which represents 71.1% of the total. For the second case, all patients who at any time were admitted to the intensive care unit were labeled with a class 1, and the rest with a class 0. In this case, only 300 patients belonged to the class 1, representing 2.9% of the total, with 10,154 patients belonging to the class 0, which represents 97.1% of the total. As can be seen, the sample data set is highly unbalanced.

## Machine learning pipeline for the classification of CoVid-19 patients

**Fig. 2:**
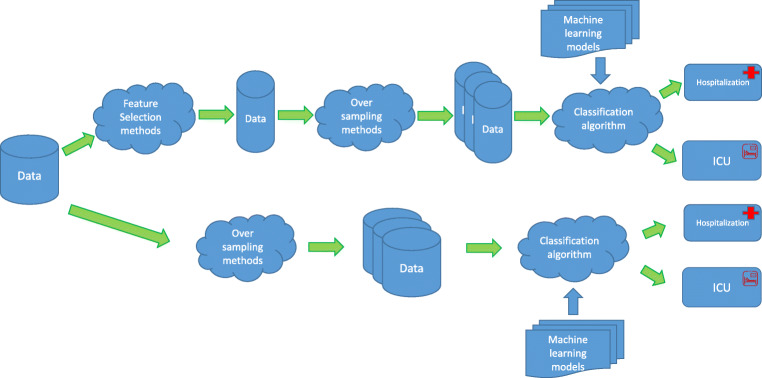
Developed machine learning pipeline

To deal with the two classification problems mentioned in the previous section, a machine learning pipeline (Fig. 2) was developed consisting of three stages:

- Feature selection.
- Over-sampling.
- Supervised classification.

### Feature selection methods

Feature selection is a preprocessing step aimed to determine the features (or variables) which are relevant for a correct classification of the data (in this case to decide if patients will be hospitalized or admitted to the ICU) and discard those features that are irrelevant or redundant [13]. For this study the Correlation-based Feature Selection (CFS) method [15] was employed. It is a multivariate filter that chooses subsets of attributes uncorrelated among them but showing a high correlation with the class. This feature selection method returns a subset of features, so there is no need to establish a threshold on the feature relevance.

The goal of this stage in the pipeline is to reduce the input dimension (833 variables) by eliminating those variables potentially less relevant to the classification problem. This could help when classifying to obtain a more robust model with better generalization power.

Data transformation techniques for feature extraction were discarded since, in this type of medical problems, we consider it very important to provide feedback and explanation to the physicians about the factors that are related to patient non-ICU and ICU admission and therefore it is mandatory to keep the original meaning of the features.

### Oversampling methods

One problem with the data set that is being handled in this research is that it has a significant imbalance between classes, i.e., the classes are not represented equally. This is especially pronounced in the case of patients who will require intensive care, since the negative class (class 0) is much more represented: 97.1% of the data. This situation is problematic for most of the machine learning methods, as they have a bias towards classes which have higher number of instances. They tend to only predict the majority class data as the features of the minority class are treated as noise in the data and thus are often ignored. As a result, on those situations there is a high probability of misclassification of the minority class as compared to the majority class. To overcome this problem, one of the most common techniques to apply is oversampling. Oversampling is used to adjust the class distribution of a data set and the goal is to balance classes in the training data before providing the data as input to the machine learning algorithm (data preprocessing). Oversampling involves introducing a bias to select or produce more samples from one class (the minority one) than from another, in order to compensate for an imbalance that is already present in the data. The most naive strategy is to generate new samples by duplicating some of the original samples of the minority class (randomly sampling with replacement). However, there are others, more sophisticated and generally better performing methods, that generate new samples by interpolation. In this research we have used the following two:

- SMOTE (Synthetic Minority Over-sampling Technique) [7] is an oversampling approach in which the minority class is over-sampled by creating “synthetic” examples. These synthetic examples cause classifiers to create larger and less specific decision regions. This approach can improve the accuracy of classifiers for a minority class.
- ADASYN [14] uses a weighted distribution for different minority class examples according to their level of difficulty in learning, where more synthetic data is generated for minority class examples that are harder to learn compared to those minority examples that are easier to learn. As a result, it improves learning with respect to the data distributions in two ways: (1) reducing the bias introduced by the class imbalance, and (2) adaptively shifting the classification decision boundary towards difficult examples.

The two methods have been configured as follows: the desired ratio of the number of samples in the minority class over the number of samples in the majority class after resampling was set to 1; and the number of nearest neighbours used to construct synthetic samples was set to 5.

### Supervised classification methods

**Fig. 3:**
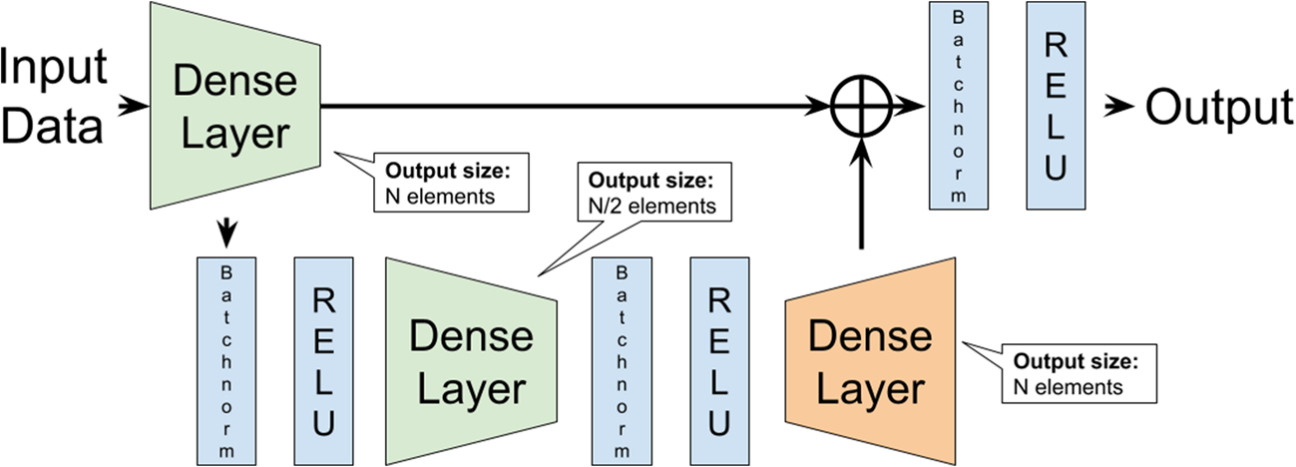
Deep Network residual block architecture

To carry out the classification task using supervised learning, different alternatives were tested to determine which would be the best from a performance point of view. To do this, the following classification methods were used, starting from basic models with linear discriminatory capacity to more complex ones with non-linear behavior:

- Logistic Regression (LR), is part of a category of statistical models called generalized linear models. The goal of logistic regression is to correctly predict the category of outcome for individual cases using the most parsimonious model. To accomplish this goal, a model is created that includes all predictor variables that are useful in predicting the response variable. Logistic regression finds a “best fitting” equation using a maximum likelihood method, which maximizes the probability of getting the observed results given the fitted regression coefficients [22].
- Multinomial Logistic Regression (MLR), is a simple extension of binary logistic regression. Like binary logistic regression, multinomial logistic regression uses maximum likelihood estimation to evaluate the probability of categorical membership.
- K Nearest Neighbor (KNN), is a supervised classification technique that classifies examples based on the ones that are most similar to them. The idea is to memorize the training set and then to predict the label of any new instance on the basis of the labels of its *k* closest neighbors in the training set. The rationale behind such a method is based on the assumption that the features that are used to describe the domain points are relevant to their labelings in a way that makes close-by points likely to have the same label [2].
- Support Vector Machine (SVM), is a supervised classification technique that works by nonlinearly projecting the training data in the input space to a feature space of higher (infinite) dimension by the use of a kernel function. This can potentially result in a linearly separable data set that can be easily discriminated by a linear classifier. In many instances, classification in high dimension feature spaces results in overfitting in the input space; however, in SVMs, overfitting is controlled through the principle of structural risk minimization [33]. The empirical risk of misclassification is minimized by maximizing the margin between the data points and the decision boundary [20]. SVM was used with lineal and nonlineal kernels (Radial Basis Function, RBF).
- AdaBoost (Adaptive Boosting), is a meta-estimator that begins by fitting a classifier on the original data set and then fits additional copies of the classifier on the same data set but where the weights of incorrectly classified instances are adjusted such that subsequent classifiers focus more on difficult cases [12]. The base classifier used in this work was a Decision Tree.
- Bagging, is an ensemble meta-estimator that fits base classifiers each on random subsets of the original data set, drawn with replacement, and then aggregates their individual predictions (either by voting or by averaging) to form a final prediction. It can typically be used as a way to reduce the variance of a black-box estimator, by introducing randomization into its construction procedure and then making an ensemble out of it [5]. In this case, the base classifier used was a SVM.
- Random Forest (RF), is a meta estimator that fits a number of decision tree classifiers on various sub-samples of the data set and uses averaging to improve the predictive accuracy and control overfitting [6].
- Multilayer Perceptron (MLP), is one of the most commonly used artificial neural network classification models [4].
- Deep Network, is a custom made residual network [16], composed of a series of multiple blocks and a final linear classifier. The block architecture is described in Fig. 3. The output size is controlled by a *N* parameter. Its value is decreased by half in the next block.

For hyperparameter optimization a grid search algorithm was applied, which is simply an exhaustive searching through a manually specified subset of the hyperparameter space of the learning algorithms. This grid search has been guided by cross-validation on the training set. Appendix A shows the hyperparameter range used for finding the best hyperparameter combination of each classification algorithm. It also includes the best tunning option for each of the algorithms in terms of the F1-score for the Hospitalization and ICU cases.

## Results

First, we will define some basic components of the metrics that we will use to evaluate classification models (notice that the data set contains only people diagnosed with CoVid-19):

- True positives (TP): number of people who have been hospitalized (or admitted to the ICU) and the method did predict that it would be necessary.
- False positives (FP): number of people who have not been hospitalized (or admitted to the ICU) but the method did identify that it would be necessary.
- True negatives (TN): number of people who have not been hospitalized (or admitted to the ICU) and the method predicted that it would not be necessary.
- False negatives (FN): number of people who have been hospitalized (or admitted to the ICU) and the method predicted that it would not be necessary.

The performance of the machine learning methods was evaluated using a 10-fold cross-validation and the following metrics were obtained from the test sets:

- Accuracy: is the number of correct global assessments, defined as the ratio (TP+TN)/(TP+TN+FP+FN).
- Sensitivity: ratio of the positive cases (those who require hospitalization or admission to the ICU) correctly identified divided by the total number of positive cases, defined as TP/(TP+FN).
- Specificity: ratio of the negative cases (those who does not require hospitalization or admission to the ICU) correctly identified divided by the total number of negative cases, defined as TN/(TN+FP).
- Area under the curve (AUC) of the receiver operating characteristic (ROC). A ROC curve is a graphical representation of sensitivity versus 1-specificity for a binary classifier system as the discrimination threshold is varied. The AUC area has a value between 0.5 and 1, where 1 represents a perfect diagnostic value and 0.5 is a test without diagnostic discriminatory capacity.
- Precision: ratio of the positive cases (those who require hospitalization or admission to the ICU) correctly identified divided by the total number of positive cases identified by the system, defined as TP/(TP+FP).
- F1-score: is a measure of a test’s accuracy. It is calculated as the harmonic mean of precision and sensitivity, defined as 2*TP/(2TP+FP+FN).

In order to verify whether the differences observed between the models in the 10-fold cross-validation were statistically significant, we applied the Kruskal-Wallis statistical test. It is a non-parametric version of ANOVA and tests the null hypothesis that the population median of all of the groups are equal. When the null hypothesis was rejected, we performed post-hoc comparisons between methods, using the Tukey’s honestly significant difference (HSD) as the multiple comparison test. In all cases we used a significance level of 0.05.

The following sections show the comparative results in each scenario (hospitalization and ICU) taking into account the following input variable configurations:

- Training the classifiers with all the variables (833): Age, Gender and the 831 binary variables of the patient’s medical history.
- Training the classifiers using only the main variables automatically selected by the feature selection method.
- Training with feature selection and oversampling.

### Problem 1: Classification of hospitalization for CoVid-19 patients

#### Classifiers trained with the original data set (833 variables)

**Fig. 4:**
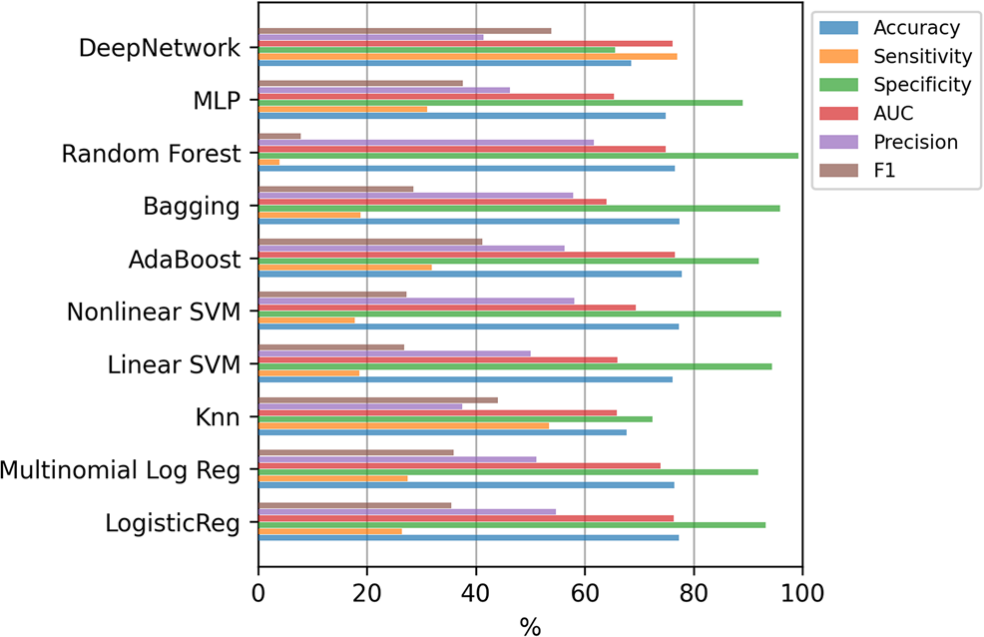
Hospitalization case. Results of the classifiers using all the variables (833)

Figure 4 shows the results of the mean value of the error evaluation metrics for the classification methods using all the variables. Although all the models obtain high values in accuracy and specificity, the values are substantially reduced in the case of sensitivity or precision. Thus, machine learning methods tend to classify all the examples in the majority class, that is, they tend to decide no hospitalization even when the patient should have been hospitalized. Due to this, throughout the work, we have decided to adopt as a criterion to determine which is the best model, to select the one that presents the highest F1-score as it is a good metric to balance sensitivity and precision. Therefore, analyzing the F1-score, it can be seen that the best model is the Deep Network. Applying the Kruskal-Wallis test, the p-value (4.3 × 10^− 15^) confirms that the differences between the methods are statistically significant. In addition, applying the Tukey’s test we verified that Deep Network has significant differences with the rest of the methods. The results of this test, comparing only the best method (Deep Network) to the others, are included in Table 4 of Appendix B.

**Fig. 5:**
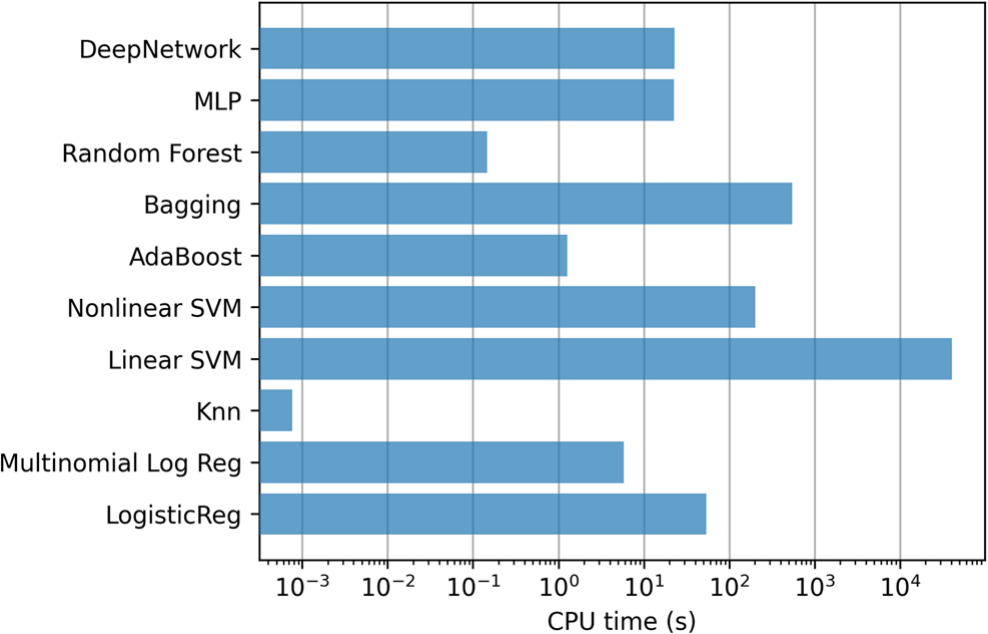
Hospitalization case. Training time of the classifiers using all the variables (833)

In addition, Fig. 5 contains the mean training time, using a logarithmic scale, for the methods in each step of the 10-fold. Clearly the most computationally expensive model is linear SVMs, which require several hours to train only one of the 10-fold steps.

#### Classifiers trained with the most relevant features

When the feature selection method was applied, 12 relevant variables were obtained for this classification problem. These variables are:

- Age.
- Gender.
- Non-insulin dependent diabetes mellitus.
- Benign prostatic hypertrophy.
- Other diagnostic procedures.
- Hydrocele.
- Viral pneumonia.
- Therapeutic advice / therapeutic listening.
- Urgent / frequent urination.
- Abnormal white blood cells.
- Other disorders of lipid metabolism.
- Heart failure.

**Fig. 6:**
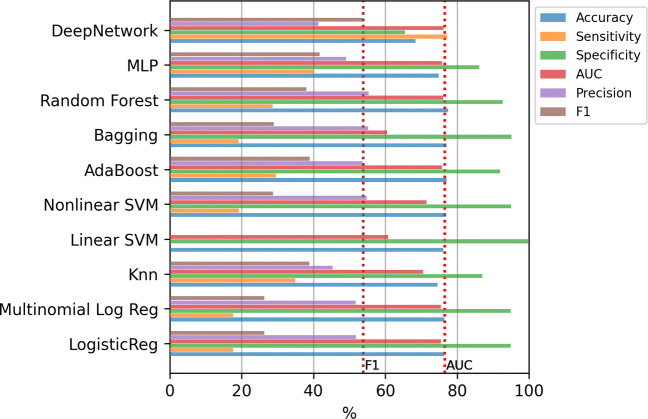
Hospitalization case. Results of the classifiers using top-12 relevant variables

Figure 6 shows the results of the mean value of the error evaluation metrics for the classification methods using the selected features. For reference, two dashed vertical lines are included that contain the best AUC and F1-score values obtained in the previous experiment (with all variables). As can be seen, in none of the cases is possible to surpass the best model obtained with all the variables.

**Fig. 7:**
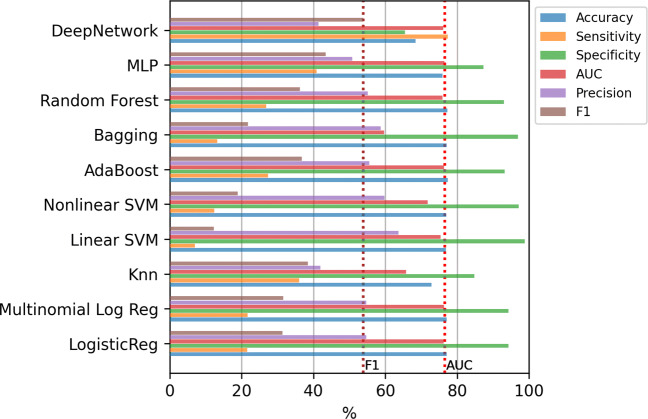
Hospitalization case. Results of the classifiers using only Age and Gender variables

As can be seen in the list of selected variables, the feature selection method places in the same category generic variables (such as Therapeutic advice / therapeutic listening) together with variables that have been shown to have certain impact on the evolution of COVID, such as Heart failure. This fact made us wonder to which extent the information on patients collected in a registry as large as 833 variables is relevant in itself for this problem. For this reason, an additional experiment was carried out, using only the two most relevant variables according to the feature selection method applied: Age and Gender. These two variables were selected in all the folds of the cross-validation procedure of the CFS method. Figure 7 contains the results for this case. The results obtained are very similar to those obtained with the 12 most relevant variables, so clearly these two variables are the ones that contain the most discriminatory information.

**Fig. 8:**
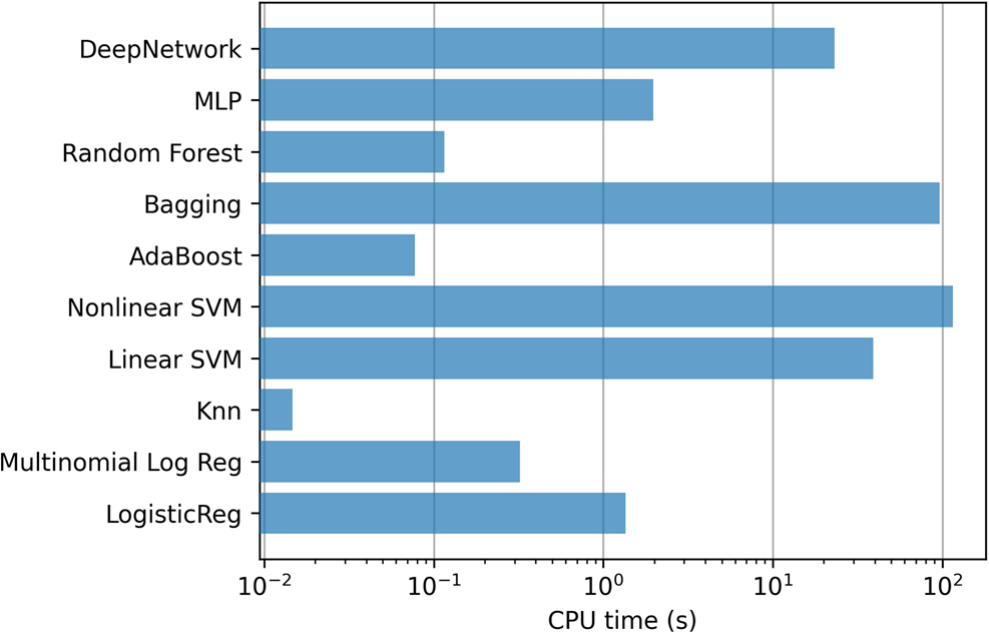
Hospitalization case. Training time of the classifiers using top-12 relevant variables

**Fig. 9:**
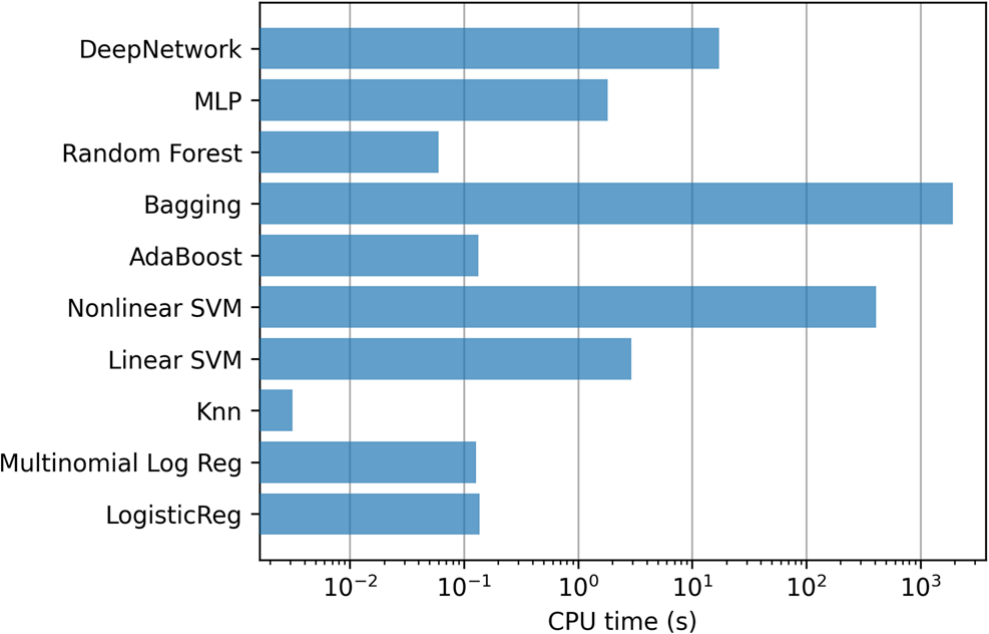
Hospitalization. Training time of the classifiers using using only Age and Gender variables

Figures 8 and 9 contain the mean training time for the methods in each step of the 10-fold. In this case, the most computationally expensive models are SVMs, Bagging and Deep Network although, having fewer input variables, computing time is reduced by an order of magnitude with respect to the experiment with all variables.

#### Classifiers trained with oversampling

**Fig. 10:**
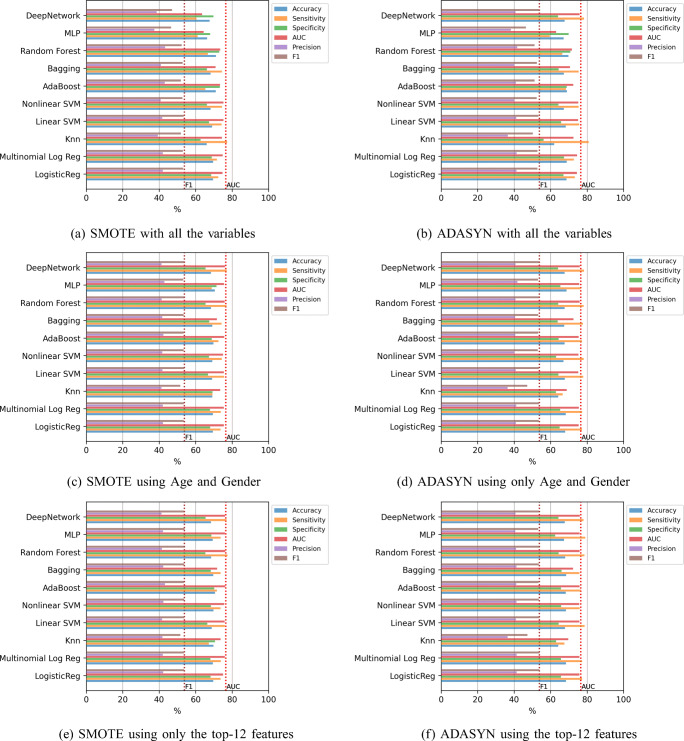
Hospitalization case. Results of the classifiers using oversampling

The last experiment consists of the incorporation of oversampling to alleviate the imbalance of the classes. Figure 10 contains the results of the mean value of the error evaluation metrics for the classification methods using SMOTE and ADASYN in three scenarios: with all the variables, using only age and gender, and using the top-12 features. The dashed vertical lines which are included, contain the best AUC and F1-score values obtained in the previous experiments. In all cases, except in the case of of the Deep Network, the F1-score results are improved significantly, since an improvement of around 10% is obtained compared to the results without oversampling. In this case the best result was obtained by the Random Forest using SMOTE and the top-12 features, with a value of 76.17% in AUC and 54.12% in F1-score, although applying the statistical test the differences are not significant with any of the other methods except with KNN.

### Problem 2: Classification of the need of ICU for CoVid-19 patients

The second issue analyzed tries to determine the possibility that a patient recently diagnosed will potentially need intensive care in the future. It is important to recall that in this case, classes are much more unbalanced (2.9% vs. 97.1%) than in the previous one.

#### Classifiers trained with the original data set (833 variables)

**Fig. 11:**
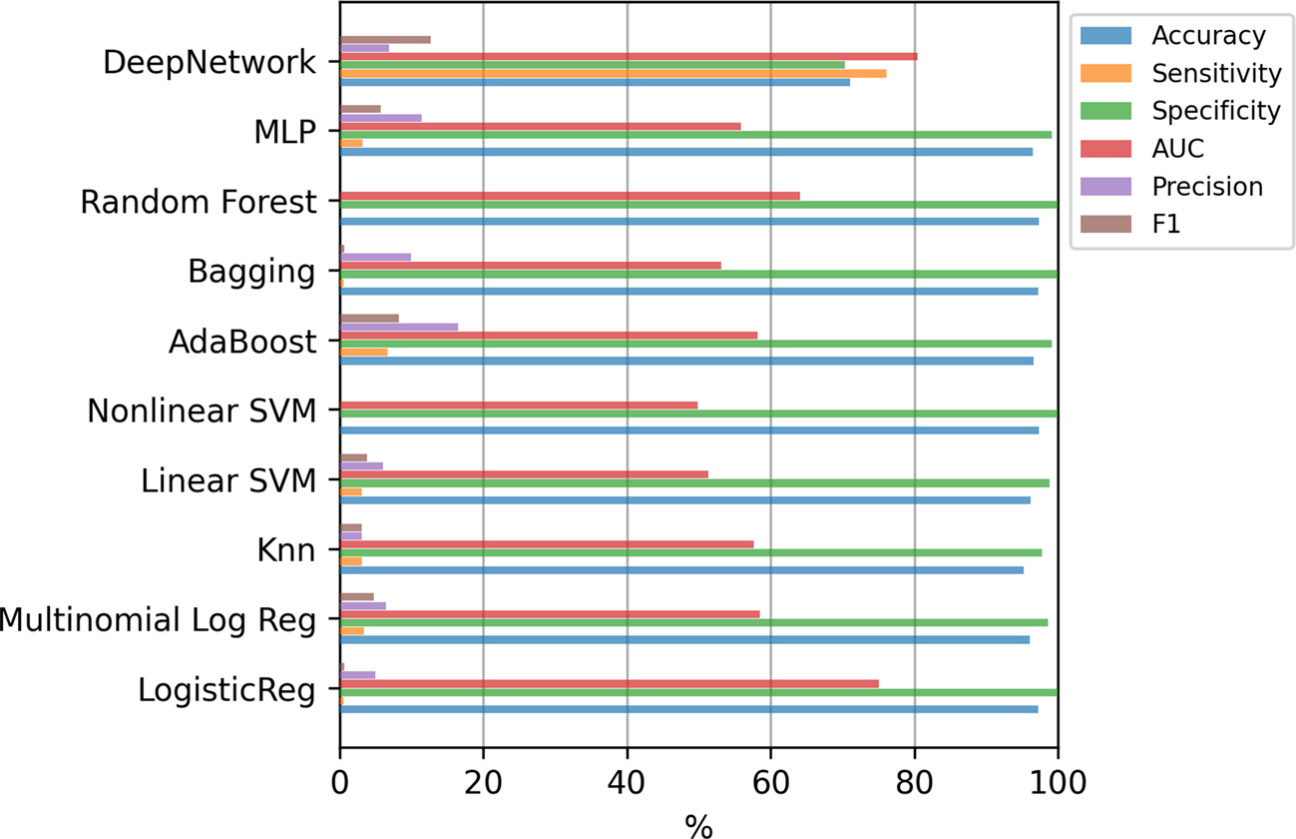
ICU case. Results of the classifiers using all the variables (833)

Figure 11 shows the results for the classifiers using all the 833 variables. Machine learning methods clearly tend again to classify all the examples as the majority class, that is, deciding that the patient will not potentially need intensive care in the future. Notice that if the values of any metric is zero, the corresponding bar does not appear in the figure. In this case, the Deep Network obtains the best results both in AUC and in F1-score. Applying the Kruskal-Wallis test, the p-value (3.5 × 10^− 9^) confirms that the differences between the methods are statistically significant. However, applying the Tukey’s test, the differences of the Deep Network is not statistically significant compared to the AdaBoost. The results of this test, comparing only the best method (Deep Network) to the others, are included in Table 5 of Appendix B.

**Fig. 12:**
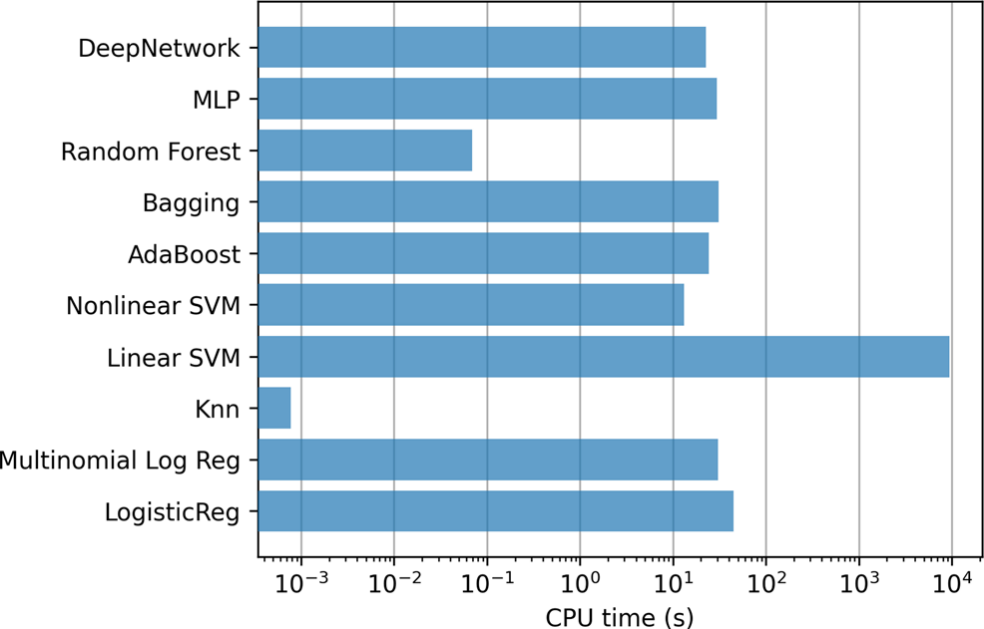
ICU case. Training time of the classifiers using all the variables (833)

In addition, Fig. 12 contains the mean training time for the methods in each step of the 10-fold. Again, the most computationally expensive model is the linear SVM, which require several hours to train each model.

#### Classifiers trained with the most relevant features

Applying the feature selection method, 20 relevant medical history variables were selected for the ICU case. These variables are the following:

- Age.
- Gender.
- Dependence.
- Cardiac ischemia with angina.
- Obesity.
- Unspecified liver diseases.
- Non-insulin dependent diabetes mellitus.
- Prostate signs / symptoms.
- Benign prostatic hypertrophy.
- Cholecystitis / cholelithiasis.
- Abdominal hernias.
- Other unspecified therapeutic / minor surgical procedures.
- Heart pressure.
- Problems with the health system.
- Clarification/discussion about reason for consultation/demand.
- Physical medicine / rehabilitation.
- Medication/application/prescription/renewal/injectables.
- Administrative procedure.
- Genital herpes, in women.
- Female genital pain.

**Fig. 13:**
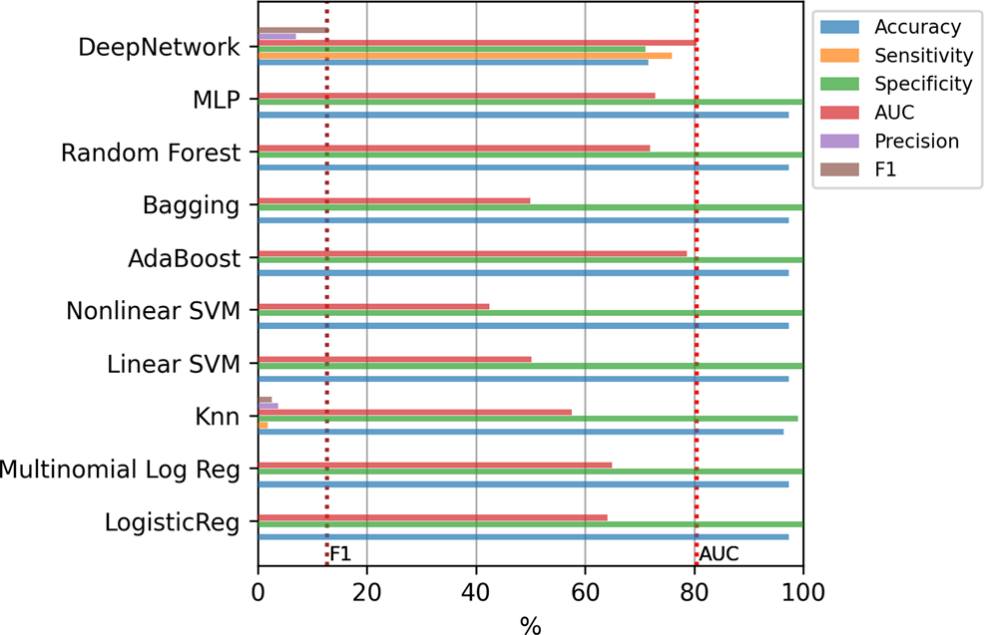
ICU case. Results of the classifiers using top-20 relevant variables

Figure 13 shows the results only using these relevant variables. Again, the two dashed vertical lines contain the best AUC and F1-score obtained in the previous experiment (with all variables). In this case, again the Deep Network is the one that obtains the best results. Applying the Kruskal-Wallis test, the p-value (9.04 × 10^− 11^) confirms that the differences between the methods are statistically significant. Besides, using the Tukey’s test we verified that the Deep Network has significant differences with the rest of the methods, except with the AdaBoost and KNN. The results of this test, comparing only the best method (Deep Network) to the others, are included in Table 6 of Appendix B.

**Fig. 14:**
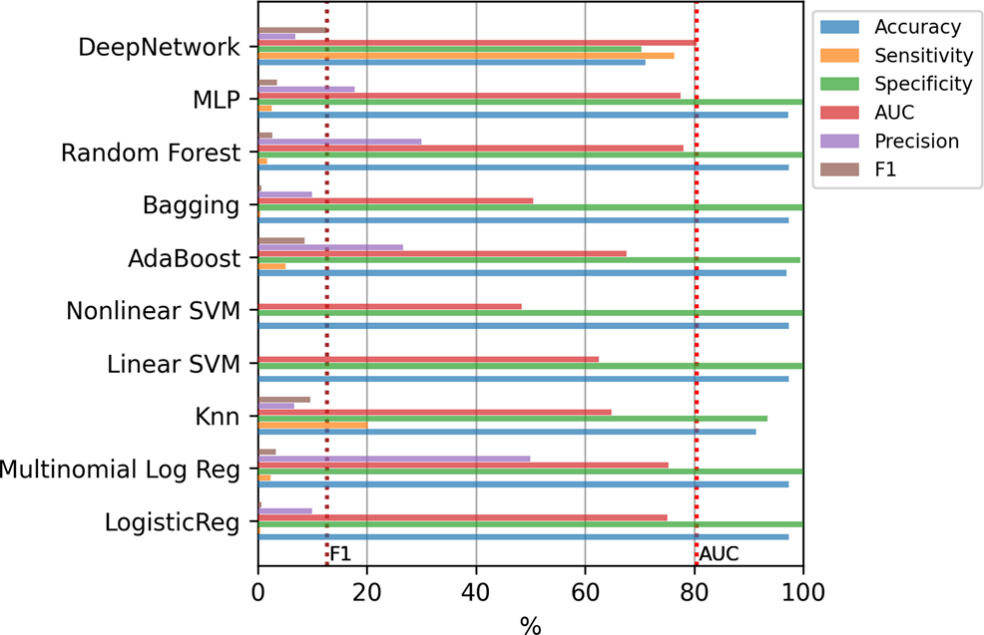
ICU case. Results of the classifiers using only Age and Gender variables

In addition, Fig. 14 shows the results using only age and gender variables. As can be seen in this case, the results are generally substantially worse than using the selected variables, since although in some cases the AUC value is improved or the accuracy is very high, it is at the cost of having a precision equal to zero.

**Fig. 15:**
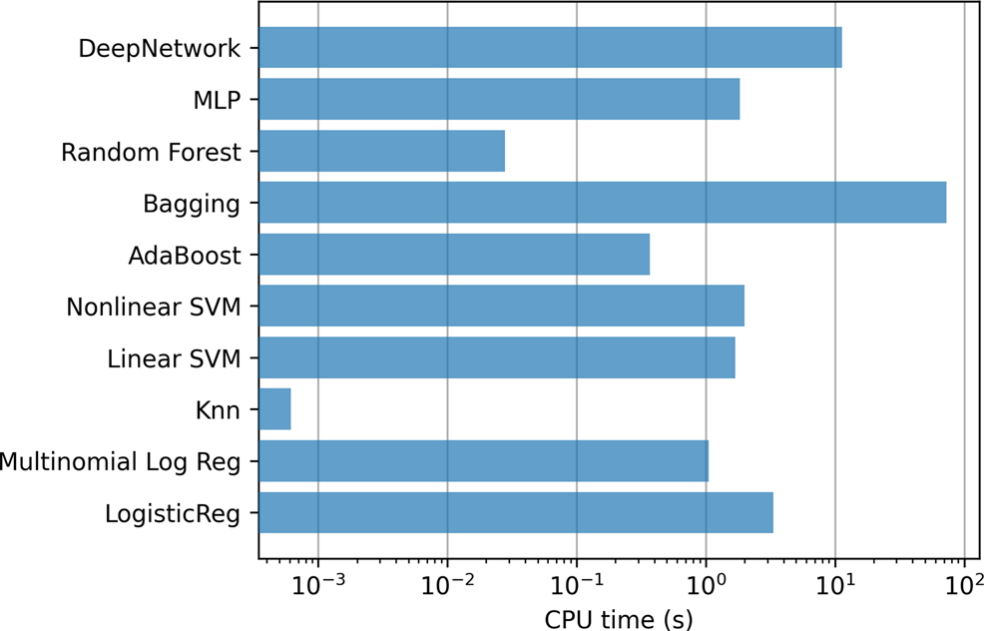
ICU case. Training time of the classifiers using top-20 relevant variables

**Fig. 16:**
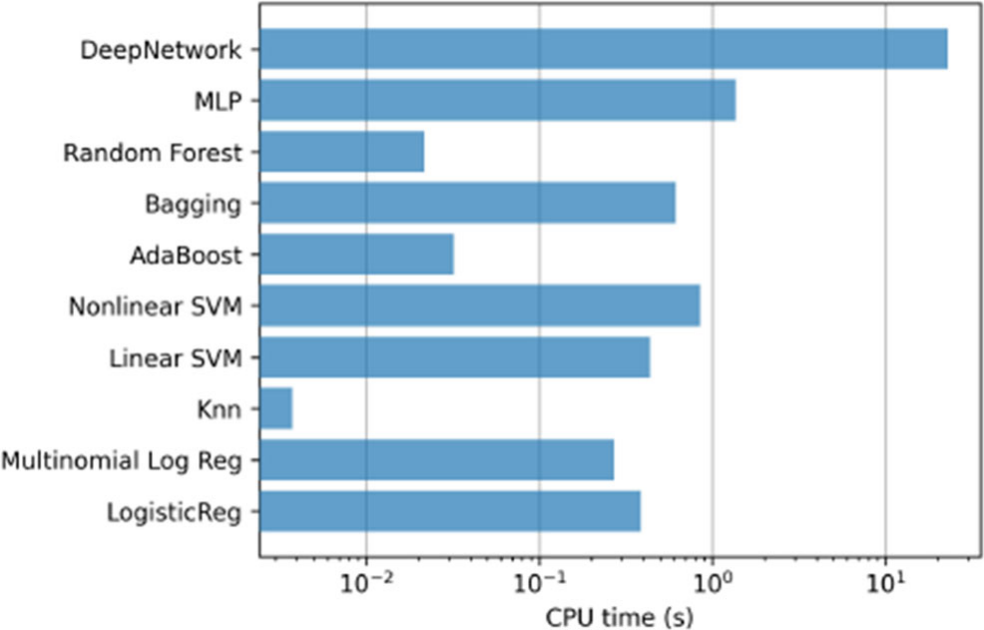
ICU case. Training time of the classifiers using using only Age and Gender variables

Figures 15 and 16 contain the mean training times for these two last experiments.

#### Classifiers trained with oversampling

**Fig. 17:**
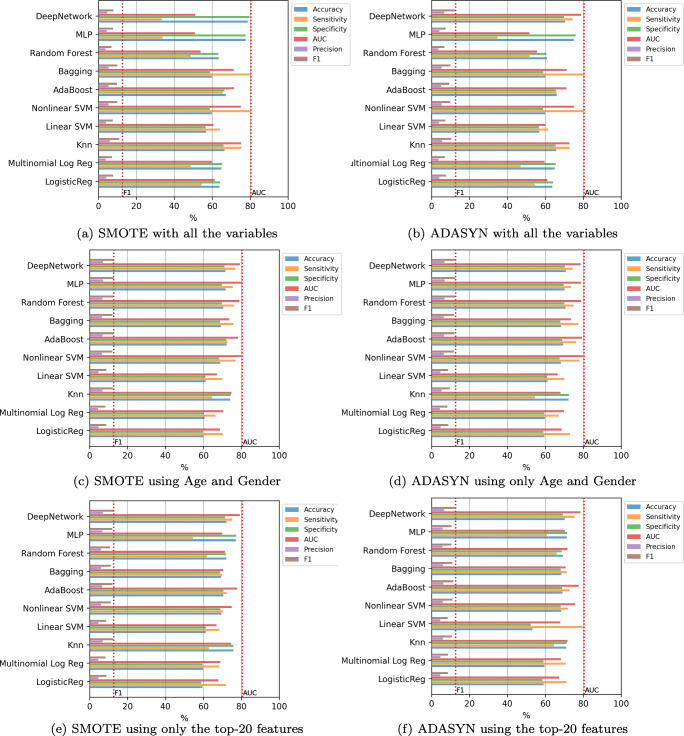
ICU case. Results of the classifiers using oversampling

The last experiment includes oversampling to alleviate the imbalance of the classes. Figure 17 contains the results of the mean value of the error evaluation metrics for the classification methods using SMOTE and ADASYN in three scenarios: with all the variables, using only age and gender, and using the top-20 features. The dashed vertical lines show the best AUC and F1-score values obtained in the previous experiments. In most of the cases, the F1-score results are improved by just over 3% compared to the results obtained without oversampling. In this case the best result was obtained by the Deep Network using SMOTE and only age and gender variables, with a value of 79.27% in AUC and 12.92% in F1-score. Nevertheless, the statistical test shows that the differences are not significant with any of the other methods except with LR, MLR and Linear SVM.

In this case, it has been found experimentally that using oversampling the variables of the medical history do not provide additional information for the automatic classification of CoVid-19 patients and only age and gender seem to be the determining factors, a founding concordant with the results obtained also by previous authors, as in the work by Nemati et al. [23], in which both variables were the most accurate predictors for 6 different algorithms.

## Conclusions

In this work, we analyzed the capability of several state of the art machine learning methods to predict whether patients diagnosed with CoVid-19 will need hospital or intensive care assistance during the course of their illness. Unlike the work by Cheng et al. [8] that have used a smaller data set from only one hospital, a data set of 10,454 patients from several hospitals in Galicia was used. Each patient was characterized by 833 variables representing the patient’s medical history besides their age and gender. Using all available features, these approaches achieved AUC values of 76,11% and 80.43% for predicting the need of hospital and ICU admissions, respectively. Although the AUC value is better in the case of the ICU, observing the F1-score values it can be seen that it is at the cost of a greater imbalance between sensitivity and precision. Over the different approaches tested, those using oversampling as a preprocessing phase offered the best results in general. The use of feature selection and oversampling allows to increase the best F1-scores from 53.84 % and 12.71% to 54.12% and 12.91%, respectively, for the two classification problems. In addition, it allows all models to improve their F1-score values significantly.

Comparing the results with the ones by Schwab et al. [31], they obtain better AUC than the ones presented in this research, but at the cost of requiring a complete blood analysis of each patient, which has the disadvantage of the economic cost required for it to be applicable to decision-making that affects a large population. In this work, we only use information already available by the health system: demographic and clinical data of each patient. The findings of our study are similar also to the results of the work described in Nemati et al. [23]. In this latter, 1,182 patients from a curated data set publicly available, with data mostly extracted from national health reports and online resources, released mainly by state/local health officials and hospitals of different countries (available in Xu et al. [35]), were examined using different statistical and machine learning methods. Their analysis concluded that a boosting model was the best predictor for discharging time of patients, and that the best results were obtained using only age and gender as model features.

In view of the experimental results obtained on the data set of patients diagnosed with CoVid-19, the following conclusions are obtained:

- If oversampling is not used, the best method is the Deep Network, with statistically significant differences with respect to the other models.
- Using oversampling, better results are obtained and, in this case, most of the models have a similar behavior between them. In this situation the Deep Network is no longer the best model.
- Of all the variables analyzed, to predict whether a patient diagnosed with CoVid-19 will need hospital or ICU care, the most relevant variables are age and gender.
- The rest of the variables recorded in the patient’s medical history in the available data apparently do not provide additional information that is decisive to allow a better discrimination of the sample than that achieved using only age and gender.

As already mentioned, the data available for this study come from several hospitals in a wide region of Spain and constitute a considerable registry of patients and variables that describe their clinical history. The fact that, using all this data, we have obtained similar good results using only age and gender leads us to draw two conclusions. First, to improve the prediction, it will be necessary to expand the clinical history to include more specific variables in relation to COVID-19, such as results of laboratory tests or X-rays. However, this information is not always available for the entire population of a country and acquiring it would imply significant additional costs for the health system. A second conclusion is that the developed system will allow governments and health managers to forecast the status of CoVid-19 patients by simply using the demographic data of their region, almost always available, without entailing any additional expense. Besides, health managers could predict the demand for hospital and ICU beds based on various hypothetical scenarios regarding the incidence of infections in the population, for instance, to answer questions such as: Which will be the demand for ICUs or hospital beds in my region if a certain percentage of the entire population was infected?

## Data Availability

The data are property of the regional government of Galicia.

## Appendix A

This appendix contains all the combination of hyperparameters used for each model in the grid search. Also, it includes the best hyperparameter tunning for each model in the Hospitalization and ICU cases. For the different models, the C parameter (LR, MLR, SVM Linear, SVM RBF), number of neighbors (knn), number of trees and their depth (AdaBoost, Bagging, RF) and number of neurons are reported.

**Table 1:** Hyperparameter optimization

Classifier	Parameter = Values
DeepNetwork	*#* neurons per block, layers per block = 4
LR	C = [0.001, 0.01, 0.1, 1, 10, 100, 1000],
	Optimiz. alg. = Stochastic Gradient Descent, Regularization = L2
MLR	C = [0.001, 0.01, 0.1, 1, 10, 100, 1000], Optimiz. alg. = Newton conjugate gradient
	Regularization = L2
Knn	*#* neighbors = [3, 5, 7, 9, ..., 23], Euclidean distance, Uniform weights
SVM lin.	C = [0.001, 0.01, 0.1, 1, 10, 100, 1000], gamma = 1/(*#* features)*var(X)
SVM RBF	C = [0.001, 0.01, 0.1, 1, 10, 100, 1000], gamma = 1/(*#* features)*var(X)
AdaBoost	Decision Tree classifier, max. depth = [1, 2, 3, 4, 5, 6, 7, 8, 9, 10],
	*#* trees = [10, 20, 30, 40, 50, 60, 70, 80, 90, 100]
Bagging	SVM RBF classifier, C = [0.001, 0.01, 0.1, 1, 10, 100, 1000],
	*#* classifiers = [10, 20, 30, 40, 50, 60, 70, 80, 90, 100],
	gamma = 1/(*#* features)*var(X)
RF	max depth = [1, 2, 3, 4, 5, 6,7, 8, 9, 10],
	*#* trees = [10, 20, 30, 40, 50, 60, 70, 80, 90, 100]
MLP	hidden layers = 2, neurons = [10, 20, 30, 40, 50, 60, 70, 80, 90, 100],
	activation = relu, solver = adam, alpha = 0.0001,
	learning rate = constant, max iter = 1000

**Table 2:** Hospitalization case: Hyperparameter best tunning for each model for F1-score

	All the variables			Age & gender			Top-12 features		
	None	ADASYN	SMOTE	None	ADASYN	SMOTE	None	ADASYN	SMOTE
DeepNetwork	[256,128,64]	[256,128,64,32,16]	[256,128,64,32]	[512,256]	[128,64]	[128,64,32,16]	[512,256,128]	[128,64,32,16,8]	[128,64,32,16]
LR	1000	0.001	0.001	1	0.01	0.1	10	10	0.001
MLR	10	0.001	0.001	1	0.01	0.1	1	1	10
Knn	3	21	21	7	21	21	3	21	21
SVM lin.	100	0.001	0.001	0.001	0.001	0.01	10	100	10
SVM RBF	1000	1	1	1000	1000	1000	1000	1000	1000
AdaBoost	[10,2]	[10,2]	[10,2]	[10,1]	[10,2]	[10,2]	[20,2]	[10,2]	[10,2]
Bagging	[30,1000]	[20,1]	[80,1]	[60,1000]	[30,1000]	[50,1000]	[10,1000]	[30,1000]	[40,1000]
RF	[10,10]	[80,10]	[40,9]	[20,4]	[60,3]	[90,4]	[40,10]	[40,6]	[50,7]
MLP	40	80	10	80	20	30	50	10	10

**Table 3:** ICU case: Hyperparameter best tunning for each model for F1-score

	All the variables			Age & gender			Top-20 features		
	None	ADASYN	SMOTE	None	ADASYN	SMOTE	None	ADASYN	SMOTE
DeepNetwork	[128,64,32]	[384]	[384]	[384,512]	[384]	[256,128,64,32,16]	[384]	[384,512,256,128,64]	[256]
LR	10	0.001	0.001	0.001	0.001	0.001	1	0.001	0.001
MLR	1000	0.001	0.001	0.001	0.001	1	100	0.001	0.001
Knn	3	19	21	3	3	13	3	21	21
SVM lin.	100	0.001	0.001	0.001	0.01	0.01	0.001	0.001	0.01
SVM RBF	0.001	1	1	0.001	100	100	0.001	100	1000
AdaBoost	[80,4]	[10,1]	[10,1]	[10,1]	[20,1]	[10,2]	[50,4]	[10,1]	[10,1]
Bagging	[10,1000]	[10,1]	[40,1]	[10,0.001]	[10,100]	[10,100]	[10,1000]	[10,100]	[50,1000]
RF	[10,1]	[10,8]	[10,10]	[10,1]	[100,5]	[20,4]	[10,10]	[80,9]	[20,10]
MLP	90	40	80	10	40	20	80	30	50

## Appendix B

This appendix contains the results of the multiple comparison test (Tukey’s honestly significant difference) performed in each experiment. The last column in each table (Reject) indicates whether the statistical test has rejected the null hypothesis for each pair of classifiers.

**Table 4:** Hospitalization case: results of the statistical test using 833 variables.

Best classifier	Other classifiers	Meandif	lower	upper	Reject
DeepNetwork	Knn	− 0.0976	-0.145	− 0.0503	True
DeepNetwork	Linear SVM	-0.2703	− 0.3176	− 0.2229	True
DeepNetwork	LogisticReg	-0.1836	− 0.2309	− 0.1363	True
DeepNetwork	MLP	-0.1621	− 0.2094	− 0.1147	True
DeepNetwork	Multinomial Log Reg	− 0.1798	− 0.2271	− 0.1324	True
DeepNetwork	Nonlinear SVM	− 0.2653	− 0.3126	− 0.218	True
DeepNetwork	Random Forest	− 0.4595	− 0.5069	− 0.4122	True
DeepNetwork	AdaBoost	0.1267	0.0794	0.1741	True
DeepNetwork	Bagging	0.2534	0.2061	0.3008	True

**Table 5:** ICU case: results of the statistical test using 833 variables.

Best classifier	Other classifiers	Meandif	lower	upper	Reject
DeepNetwork	Knn	− 0.0962	− 0.1472	− 0.0452	True
DeepNetwork	Linear SVM	− 0.0891	− 0.1402	− 0.0381	True
DeepNetwork	LogisticReg	− 0.1207	− 0.1717	− 0.0696	True
DeepNetwork	MLP	− 0.0696	− 0.1207	− 0.0186	True
DeepNetwork	Multinomial Log Reg	− 0.079	− 0.13	− 0.028	True
DeepNetwork	Nonlinear SVM	− 0.1271	− 0.1782	− 0.0761	True
DeepNetwork	Random Forest	− 0.1271	− 0.1782	− 0.0761	True
DeepNetwork	AdaBoost	0.0445	− 0.0065	0.0956	False
DeepNetwork	Bagging	0.1205	0.0694	0.1715	True

**Table 6:** ICU case: results of the statistical test using the 20-top relevant features.

Best classifier	Other classifiers	Meandif	lower	upper	Reject
DeepNetwork	Knn	− 0.0309	− 0.0799	0.0181	False
DeepNetwork	Linear SVM	− 0.1272	− 0.1762	− 0.0783	True
DeepNetwork	LogisticReg	− 0.1206	− 0.1696	− 0.0716	True
DeepNetwork	MLP	− 0.092	− 0.141	− 0.043	True
DeepNetwork	Multinomial Log Reg	− 0.0937	− 0.1427	− 0.0447	True
DeepNetwork	Nonlinear SVM	− 0.1272	− 0.1762	− 0.0783	True
DeepNetwork	Random Forest	− 0.1006	− 0.1495	− 0.0516	True
DeepNetwork	AdaBoost	0.0409	− 0.0081	0.0899	False
DeepNetwork	Bagging	0.1206	0.0716	0.1696	True

## Code availability

The python code is available.
